# Reply to Nifli, A.-P. Comment on “Rosell-Cardona et al. Dietary Spray-Dried Porcine Plasma Reduces Neuropathological Alzheimer’s Disease Hallmarks in SAMP8 Mice. *Nutrients* 2021, *13*, 2369”

**DOI:** 10.3390/nu13114065

**Published:** 2021-11-13

**Authors:** Cristina Rosell-Cardona, Christian Griñan-Ferré, Anna Pérez-Bosque, Javier Polo, Mercè Pallàs, Concepció Amat, Miquel Moretó, Lluïsa Miró

**Affiliations:** 1 Department of Biochemistry and Physiology, Faculty of Pharmacy and Food Sciences, Institute for Nutrition and Food Safety, Universitat de Barcelona (UB), 08028 Barcelona, Spain; cristina.rosell@ub.edu (C.R.-C.); anna.perez@ub.edu (A.P.-B.); camat@ub.edu (C.A.); mmoreto@ub.edu (M.M.); 2 Department of Pharmacology, Toxicology, and Medicinal Chemistry, Faculty of Pharmacy and Food Sciences, Institute of Neurosciences, CIBERNED, Universitat de Barcelona (UB), 08028 Barcelona, Spain; christian.grinan@ub.edu (C.G.-F.); pallas@ub.edu (M.P.); 3 APC Europe S.L.U., 08403 Granollers, Spain; javier.polo@apc-europe.com

Thank you for your comments on our recent work of the effects of supplementation with spray-dried porcine plasma (SDP) on neuropathological markers of Alzheimer’s disease (AD) [1]. The author widely describes the use of blood and blood containing food in different countries and its use in different periods of age, but we would like to point out that SDP is a plasma product, so it does not content the blood cells fraction. Therefore, despite the fact that, as the author indicates, the consumption of raw blood and the SDP supplement improve the barrier function in the intestine [2,3] as well as food digestibility and growth [4], it should be noted that there are large differences between the two products, both in terms of the compounds they contain and in terms of safety.

At the component level, SDP is a complex mixture of many functional components such as albumin, immunoglobulins, transferrin, fibrinogen, growth factors and many other peptides, which can develop a biological activity, not only in the intestine but also at the systemic level, regardless of its nutritional value [5]. Its mechanism of action may involve the interaction of several of its functional components with cells in the body, as well as a prebiotic effect on the intestinal microbiota with anti-inflammatory effects, as observed by Moretó et al. [6].

Regarding the concern for security, it is necessary to mention a couple of aspects to consider. On the one hand, the blood used to produce SDP or serum bovine immunoglobulins (SBI), either from porcine or bovine origin, is obtained from healthy animals declared fit for human consumption after veterinary inspection by the competent authorities. This prevents the collection of blood from sick animals or animals coming from areas of OIE (World Organization for Animal Health) notifiable disease [7]. 

On the other hand, the manufacturing process of SDP and SBI has been extensively investigated to inactivate microorganism of concern for either human or animal consumption [8]. In case of African Swine Fever virus (ASFV), recent publications [9,10] demonstrated that the different steps involved in the manufacturing process of SDP or SBI (spray-drying and storage at 20 °C for 14 days) can be considered robust inactivation steps according to World Human Organization (2004) guidelines for human plasma transfusion. Furthermore, with regard to the risk of bovine spongiform encephalopathy (BSE) of prions in the collected blood, in the case of blood from pigs there is no risk of prions diseases because encephalopathies has not been established in natural conditions in commercial pigs [11] and, in the case of bovine origin, blood is not considered specific risk material according to the OIE and, in fact, blood and blood products, such as SBI or SDP, are included in the list of safe commodities like milk [12].

Although more studies are needed to understand its mechanism of action, SDP shows neuroprotective effects in the elderly population, increasing brain resilience and reducing neuroinflammation.

## Data Availability

The datasets generated during and/or analyzed during the current study are available from the corresponding author on reasonable request.
